# Impact of bone mineral density testing in the national health screening program on osteoporosis-related medical visits and fractures among women

**DOI:** 10.1007/s11657-026-01673-1

**Published:** 2026-03-25

**Authors:** Jae Hyeok Lim, Jiyeon Chun, Gyung-Joo Min, Dan Bi Kim, Jisu Ko, Eun-Cheol Park

**Affiliations:** 1 Department of Public Health, Graduate School, Yonsei University, Seoul, Republic of Korea; 2 Institute of Health Services Research, Yonsei University, Seoul, Republic of Korea; 3 Department of Preventive Medicine, Yonsei University College of Medicine, 50 Yonsei-Ro, Seodaemun-Gu, Seoul, 03722 Republic of Korea

**Keywords:** Bone mineral density, Osteoporosis, National screening, Fracture, Cohort study

## Abstract

***Summary*:**

Evaluation of the effectiveness of bone mineral density (BMD) testing within national health screening programs should consider country-specific contexts when applied to asymptomatic populations. BMD testing at age 66 in women was associated with increased osteoporosis-related medical visit and a reduction of subsequent fracture incidence. These findings suggest potential benefits of population-based screening with BMD testing.

**Purpose:**

Early detection of osteoporosis through bone mineral density (BMD) testing may help prevent future fractures. This study aimed to investigate the impact of incorporating BMD testing in South Korea’s national health screening program on outpatient visits with an osteoporosis diagnosis and fractures.

**Methods:**

We used data from the Korean National Health Insurance Service–Senior Cohort (2002–2019) and included only women aged 66 years, as specified by national screening policy, without prior osteoporosis who underwent national health screening between 2004 and 2009. Screening periods were categorized by the inclusion of BMD testing. Outcomes included osteoporosis-related medical visits within two years and incident osteoporotic fractures. Multivariable logistic regression and Cox proportional hazards regression were used to examine osteoporosis detection and fracture risk, respectively.

**Results:**

Among the 24,895 women screened, 24.7% had osteoporosis-related medical visits within two years, and 21.5% experienced fractures during follow-up. Compared to the period without BMD testing, the inclusion of BMD testing was associated with a 52% increase in osteoporosis-related medical visits (odds ratio: 1.52, 95% confidence interval [CI]: 1.42–1.62), whereas the risk of subsequent fractures was reduced by 9% (hazard ratio: 0.91, 95% CI: 0.86–0.96). These associations were more pronounced among those with low body mass index and significant during the 5–10 years of follow-up for hip and vertebral fractures.

**Conclusion:**

Nationwide implementation of BMD testing increased the medical visits for osteoporosis and was associated with a reduction in subsequent fractures. To further enhance the effectiveness of the screening program, improved post-screening management is needed.

**Supplementary Information:**

The online version contains supplementary material available at 10.1007/s11657-026-01673-1.

## Introduction

Osteoporosis is a condition characterized by reduced bone mass, impaired bone strength, and deterioration of the bone microarchitecture, leading to an increased risk of fragility fractures [1]. According to a meta-analysis in 2021, the global prevalence of osteoporosis is estimated at 18.3% [2]. In South Korea, recent data indicate that among individuals aged 50 years and older, 7.5% of men and 37.3% of women are affected by the disease [3]. Fractures related to osteoporosis are associated with increased mortality and substantial healthcare costs, making them a serious public health concern [4, 5]. Given the ongoing global trend of population aging, the burden of osteoporosis and related fractures is expected to rise further in the coming years [6].

Bone mineral density (BMD) testing is the primary method for diagnosing osteoporosis and, together with tools such as the FRAX score, is used as a screening approach to partially predict future fracture risk [7]. BMD testing is regarded as an essential and feasible strategy for detecting osteoporosis or identifying individuals at a high risk of fracture in population-based screening settings, thereby facilitating timely interventions to reduce adverse health outcomes. Recognizing these benefits, BMD testing was first introduced in 2007 as part of the National Screening Program for Transitional Ages, targeting only 66-year-old women, who were designated as a high-risk age group under the national screening policy in South Korea [8]. As the national health screening program in South Korea is designed to cover the entire population, most of the citizens who fulfill the specified age and sex criteria are deemed eligible for screening.

However, the expansion of such screening programs has not always been based on robust scientific evidence. This concern might also apply to BMD testing, as the overall program has been criticized for insufficient justification of implementation and a lack of formal evaluation regarding its effectiveness [9, 10]. International guidelines also vary with respect to osteoporosis screening recommendations. According to the U.S. Preventive Services Task Force (USPSTF), universal screening is recommended for all women aged 65 and older [11], whereas European guidelines typically advocate BMD testing only for individuals identified as high-risk following a fracture risk assessment [12, 13]. These differences underscore the need to consider country-specific contexts when evaluating the rationale for screening implementation. In particular, the high prevalence of osteoporosis among Asian women may warrant differing screening recommendations [14]. Although prior Korean studies have examined the predictive value of BMD testing for fractures and changes in disease prevalence following screening [15, 16], a comprehensive evaluation of the screening program’s effectiveness is required to determine the justification for its continued implementation [17].

Therefore, in this study, we aimed to investigate whether the implementation of BMD testing for women within the South Korean national health screening program influenced the osteoporosis-related medical visits and incidence of osteoporotic fractures, taking advantage of a natural policy shift in screening items.

## Methods

### Data

This retrospective cohort study used data from the Korean National Health Insurance Service–Senior Cohort (NHIS-SC), a population-based sample of approximately 512,000 individuals—about 8% of all adults aged ≥ 60 years who were eligible for national health insurance in 2008. The sample was selected using stratified random sampling based on sex, age, region, and income level. The NHIS-SC provides de-identified data from 2002 to 2019, including demographic information, healthcare utilization across various settings (hospitals, clinics, nursing homes, and long-term care facilities), and national health screening records. Diagnoses are recorded using ICD-10 codes according to South Korea’s national health insurance claims standards [18].

### Participants

BMD testing was included in the South Korean national screening program for transitional ages in April 2007, targeting only women aged 66 years. Accordingly, we designated 2002 and 2003 as a washout period and identified 43,376 women who underwent the national health screening at the age of 66 between 2004 and 2009. To evaluate the impact of the policy change, the period was divided into two phases: screening without BMD testing (January 2004 to March 2007) and screening with BMD testing (April 2007 to December 2009). Women with a prior history of osteoporosis at the age of 66 years were excluded before undergoing the health screening. As a result, 8704 women who had been diagnosed with osteoporosis (ICD-10 codes M80–M82) and 9254 women who had been prescribed osteoporosis-related medications—including bisphosphonates, selective estrogen receptor modulators, or calcitonin—were excluded. In addition, Medical Aid beneficiaries were excluded because information prior to 2006 was unavailable and the initiation of the general health screening program differed for this population. After these exclusions, a total of 24,895 women were included in the final analysis.

### Variables

#### Participation in national health screening with bone mineral density testing

Participants were classified based on whether they underwent the national health screening program before or after the implementation of the National Screening Program for Transitional Ages in April 2007. Those who received the screening prior to April 2007 were categorized as having undergone the program without BMD testing, whereas those screened after April 2007 were categorized as having received the program with BMD testing.

#### Osteoporosis-related medical visit and incidence of fractures

Two outcomes were assessed: (1) osteoporosis-related medical visits and (2) incident fractures. Osteoporosis detection was defined as at least one medical claim with a diagnosis code for osteoporosis (ICD-10 codes M80–M82) within two years following the index health screening date, reflecting the biennial interval of national health screenings in South Korea. Fractures were defined as new events occurring after the screening, identified using ICD-10 codes for common fracture sites associated with osteoporosis: hip (S720, S721), vertebrae (S220, S221, S320, M484, M485), distal radius (S525, S526), and humerus (S422, S423) [19]. The time from the index screening date to the first incidence of a fracture was calculated for time-to-event analyses. To account for systematic differences in index dates among participants, the follow-up period was uniformly censored at 10 years, corresponding to the maximum potential follow-up duration for individuals screened at the latest index date. Participants were followed from the index date until the earliest incidence of a fracture event, death, or the end of the 10-year follow-up period, whichever came first. Factures were further categorized by type and time period since incidence: 0–2 years, 2–5 years, and more than 5 years.

#### Covariates

Sociodemographic and health-related factors were included in the model as covariates: income level, type of medical insurance, residential region, disability, Charlson Comorbidity Index (CCI; 0, 1, and > 1), body mass index (BMI; underweight: < 18.5 kg/m^2^, normal: 18.5–23 kg/m^2^, overweight: 23–25 kg/m^2^, and obesity: > 25 kg/m^2^), physical activity, smoking status, and alcohol consumption (none, mild: two or fewer times per week, and heavy: three or more times per week). To control for underlying temporal trends, we grouped cohort years into three categories (year 1: 2004/2007, year 2: 2005/2008, year 3: 2006/2009) based on their relative position before and after the inclusion of BMD testing. CCI provides weighted score ratings (1–6) to 19 comorbid diseases, matching them with each of the 17 comorbidities based on their corresponding 1-year mortality risks [20]. Physical activity was classified based on the WHO guidelines, considering yearly survey variations: vigorous exercise inducing sweating three or more times or ≥ 150 min of moderate-intensity activity, ≥ 75 min of vigorous-intensity activity, or an equivalent combination of activity per week [21]. Data on BMI, physical activity, smoking, and alcohol consumption were sourced from health screening measurements and self-reported records. For participants with missing values at the index screening, the corresponding variable was supplemented using information from the closest available screening date. If no such data were available, missing values were classified as a missing category.

### Statistical analyses

Descriptive statistics were used to summarize the baseline characteristics of the study population. The distributions of independent variables were presented as frequencies and percentages according to the incidence of each outcome, and group differences were assessed using the chi-square test. Time-to-fracture following the index screening was plotted using cumulative incidence curves, and differences between groups were evaluated with the log-rank test.

Associations between participation in BMD testing and subsequent medical visits for osteoporosis were examined using multivariable logistic regression. The risk of incident fractures after screening was examined using multivariable Cox proportional hazards regression. Additional analyses were conducted by type of fracture and time period since the fracture incidence. All models were adjusted for predefined covariates, and individuals with missing covariate data were excluded from the regression analysis. Subgroup analyses were conducted stratified by selected covariates, with adjustment for all other covariates except the stratifying variable. Results from the logistic regression models were reported as odds ratios (ORs) with 95% confidence intervals (CIs), and those from the Cox regression models were reported as hazard ratios (HRs) with 95% CIs. All statistical tests were two-sided, and a *p*-value of < 0.05 was considered statistically significant. Analyses were conducted using SAS software, version 9.4 (SAS Institute Inc., Cary, NC, USA).

## Results

### Descriptive analysis

Table 1 summarizes the baseline general characteristics of the study population. A total of 24,895 women aged 66 years without a prior history of osteoporosis underwent national health screening between 2004 and 2009. Of these, 10,566 (42.4%) were screened before the introduction of BMD testing (April 2004 to March 2007), and 14,329 (57.6%) were screened after its implementation (April 2007 to December 2009). Among the study population, 6141 women (24.7%) visited a medical institution for osteoporosis within two years after the screening, and 5342 women (21.5%) experienced fractures during the follow-up period. Specifically, the incidence of fractures was 25.7 per 1000 person-years in the national screening group without BMD testing and 22.7 per 1000 person-years in those with BMD testing (Supplementary Table S1).

**Table 1:** General characteristics of the study population at the baseline

Variables	Total		Osteoporosis-related visit					Fractures				
			Yes		No		*P*-value	Yes		No		*P*-value
	N	%	N	%	N	%		N	%	N	%	
Total (*N* = 24,895)	24,895	100.0	6141	24.7	18,754	75.3		5342	21.5	19,553	78.5	
BMD testing							< 0.0001					< 0.0001
No	10,566	42.4	2164	20.5	8402	79.5		2419	22.9	8147	77.1	
Yes	14,329	57.6	3977	27.8	10,352	72.2		2923	20.4	11,406	79.6	
Income level							0.0270					0.1387
Low	4211	16.9	971	23.1	3240	76.9		923	21.9	3288	78.1	
Middle	9588	38.5	2409	25.1	7179	74.9		2102	21.9	7486	78.1	
High	11,096	44.6	2761	24.9	8335	75.1		2317	20.9	8779	79.1	
Residence area							< 0.0001					< 0.0001
Metropolitan	8281	33.3	1897	22.9	6384	77.1		1606	19.4	6675	80.6	
City	5793	23.3	1489	25.7	4304	74.3		1237	21.4	4556	78.6	
Rural	10,821	43.5	2755	25.5	8066	74.5		2499	23.1	8322	76.9	
Disability							0.3801					0.0019
No	23,059	92.6	5672	24.6	17,387	75.4		4895	21.2	18,164	78.8	
Yes	1836	7.4	469	25.5	1367	74.5		447	24.3	1389	75.7	
CCI							< 0.0001					< 0.0001
0	11,827	47.5	2731	23.1	9096	76.9		2417	20.4	9410	79.6	
1	7347	29.5	1902	25.9	5445	74.1		1582	21.5	5765	78.5	
> 1	5721	23.0	1508	26.4	4213	73.6		1343	23.5	4378	76.5	
Body mass index							< 0.0001					0.0004
Underweight	491	2.0	120	24.4	371	75.6		130	26.5	361	73.5	
Normal	6976	28.0	1861	26.7	5115	73.3		1566	22.4	5410	77.6	
Overweight	6506	26.1	1656	25.5	4850	74.5		1413	21.7	5093	78.3	
Obesity	10,922	43.9	2504	22.9	8418	77.1		2233	20.4	8689	79.6	
Physical activity							0.2091					0.0028
No	16,993	68.3	4177	24.6	12,816	75.4		3733	22.0	13,260	78.0	
Yes	6955	27.9	1764	25.4	5191	74.6		1406	20.2	5549	79.8	
Missing	947	3.8	-	-	-	-		-	-	-	-	
Smoking status							0.9495					< 0.0001
None	23,272	93.5	5,782	24.8	17,490	75.2		4944	21.2	18,328	78.8	
Past	188	0.8	52	27.7	136	72.3		56	29.8	132	70.2	
Current	555	2.2	136	24.5	419	75.5		153	27.6	402	72.4	
Missing	880	3.5	-	-	-	-		-	-	-	-	
Drinking							0.1906					0.0033
None	21,891	87.9	5447	24.9	16,444	75.1		4668	21.3	17,223	78.7	
Mild	1563	6.3	390	25.0	1173	75.0		337	21.6	1,226	78.4	
Heavy	549	2.2	118	21.5	431	78.5		150	27.3	399	72.7	
Missing	892	3.6	-	-	-	-		-	-	-	-	
Cohort year							0.0018					< 0.0001
Year 1	6899	27.7	1647	23.9	5252	76.1		1627	23.6	5272	76.4	
Year 2	9276	37.3	2253	24.3	7023	75.7		1956	21.1	7320	78.9	
Year 3	8720	35.0	2241	25.7	6479	74.3		1759	20.2	6961	79.8	

Among participants who underwent BMD testing within the cohort, more than half were assessed using dual-energy X-ray absorptiometry (Supplementary Table S2). According to the test results, 16.5% had normal BMD, 40.1% were classified as having osteopenia, and 43.4% were diagnosed with osteoporosis, indicating that a substantial proportion required follow-up management after screening.

Supplementary Fig. S1 displays the cumulative incidence curves of fractures according to BMD testing status. A divergence between the two groups emerged over time, and the difference was statistically significant based on the log-rank test (*p* < 0.0001).

### Multivariable regression analysis

Table 2 presents the results of logistic regression and Cox proportional hazards regression analyses for osteoporosis-related medical visits and fracture incidence, respectively. Individuals who underwent national screening without BMD testing had 52% higher odds (OR: 1.52, 95% CI: 1.42–1.62) of visiting a medical institution for osteoporosis within two years after screening than those who received BMD-inclusive screening. Conversely, their risk of fracture during the follow-up period was reduced by 9% (HR: 0.91, 95% CI: 0.86–0.96).

**Table 2:** Result of regression model for osteoporosis-related visit and fractures

Variables	Osteoporosis-related visit				Fractures			
	OR	95% CI			HR	95% CI		
BMD testing								
No	1.00				1.00			
Yes	1.52	(1.42	-	1.62)	0.91	(0.86	-	0.96)
Income level								
Low	1.00				1.00			
Middle	1.11	(1.01	-	1.21)	0.99	(0.91	-	1.07)
High	1.08	(0.99	-	1.17)	0.94	(0.87	-	1.02)
Residence area								
Metropolitan	1.00				1.00			
City	1.17	(1.08	-	1.27)	1.11	(1.03	-	1.19)
Rural	1.17	(1.09	-	1.25)	1.17	(1.10	-	1.25)
Disability								
No	1.00				1.00			
Yes	1.00	(0.90	-	1.12)	0.85	(0.77	-	0.94)
CCI								
0	1.00				1.00			
1	1.16	(1.08	-	1.25)	1.05	(0.98	-	1.12)
> 1	1.18	(1.10	-	1.27)	1.19	(1.11	-	1.27)
Body mass index								
Underweight	1.00				1.00			
Normal	1.08	(0.86	-	1.34)	0.85	(0.70	-	1.02)
Overweight	0.99	(0.79	-	1.23)	0.83	(0.69	-	1.00)
Obesity	0.87	(0.70	-	1.08)	0.76	(0.63	-	0.91)
Physical activity								
No	1.00				1.00			
Yes	0.99	(0.92	-	1.05)	0.94	(0.89	-	1.01)
Smoking status								
None	1.00				1.00			
Past	1.20	(0.86	-	1.68)	1.39	(1.05	-	1.83)
Current	0.98	(0.80	-	1.19)	1.28	(1.08	-	1.50)
Drinking								
None	1.00				1.00			
Mild	1.01	(0.89	-	1.14)	1.02	(0.91	-	1.14)
Heavy	0.94	(0.76	-	1.16)	1.29	(1.09	-	1.52)
Cohort year								
Year 1	1.00				1.00			
Year 2	0.99	(0.92	-	1.07)	0.88	(0.82	-	0.94)
Year 3	1.07	(0.99	-	1.16)	0.85	(0.79	-	0.91)

### Additional analysis

Table 3 presents the results of subgroup analyses stratified by independent variables included in the model. A stronger association was observed in subgroups defined by lower BMI. Specifically, the association was more pronounced among underweight (osteoporosis-related medical visit, OR: 1.97, 95% CI: 1.23–3.16; fractures, HR: 0.60, 95% CI: 0.41–0.90) and normal-weight individuals (osteoporosis-related medical visit, OR: 1.68, 95% CI: 1.49–1.88; fractures, HR: 0.89, 95% CI: 0.80–0.98), whereas it was attenuated among those who were overweight (osteoporosis-related medical visit, OR: 1.46, 95% CI: 1.29–1.65; fractures, HR: 0.91, 95% CI: 0.82–1.02) or obese (osteoporosis-related medical visit, OR: 1.43, 95% CI: 1.30–1.58; fractures, HR: 0.94, 95% CI: 0.86–1.02). In contrast to BMI, similar patterns were not observed for other health behavior-related variables.

**Table 3:** Results of subgroup analysis stratified by independent variables

Variables	Osteoporosis-related visit					Fractures				
	No	Yes				No	Yes			
	OR	OR	95% CI			HR	HR	95% CI		
Body mass index										
Underweight	1.00	1.97	(1.23	-	3.16)	1.00	0.60	(0.41	-	0.90)
Normal	1.00	1.68	(1.49	-	1.88)	1.00	0.89	(0.80	-	0.98)
Overweight	1.00	1.46	(1.29	-	1.65)	1.00	0.91	(0.82	-	1.02)
Obesity	1.00	1.43	(1.30	-	1.58)	1.00	0.94	(0.86	-	1.02)
Physical activity										
No	1.00	1.52	(1.42	-	1.64)	1.00	0.90	(0.85	-	0.97)
Yes	1.00	1.49	(1.31	-	1.70)	1.00	0.91	(0.81	-	1.02)
Smoking status										
None	1.00	1.53	(1.43	-	1.63)	1.00	0.91	(0.86	-	0.97)
Past	1.00	0.61	(0.27	-	1.38)	1.00	1.07	(0.60	-	1.94)
Current	1.00	1.57	(1.02	-	2.42)	1.00	0.64	(0.46	-	0.90)
Drinking										
None	1.00	1.55	(1.45	-	1.65)	1.00	0.90	(0.85	-	0.95)
Mild	1.00	1.24	(0.95	-	1.60)	1.00	0.92	(0.73	-	1.16)
Heavy	1.00	1.22	(0.77	-	1.97)	1.00	1.10	(0.77	-	1.56)
Cohort year										
Year 1	1.00	1.52	(1.35	-	1.72)	1.00	0.81	(0.82	-	1.01)
Year 2	1.00	1.45	(1.31	-	1.61)	1.00	0.94	(0.85	-	1.03)
Year 3	1.00	1.59	(1.43	-	1.76)	1.00	0.87	(0.79	-	0.96)

Adjusted all covariates except for each stratified variable

**Fig. 1:**
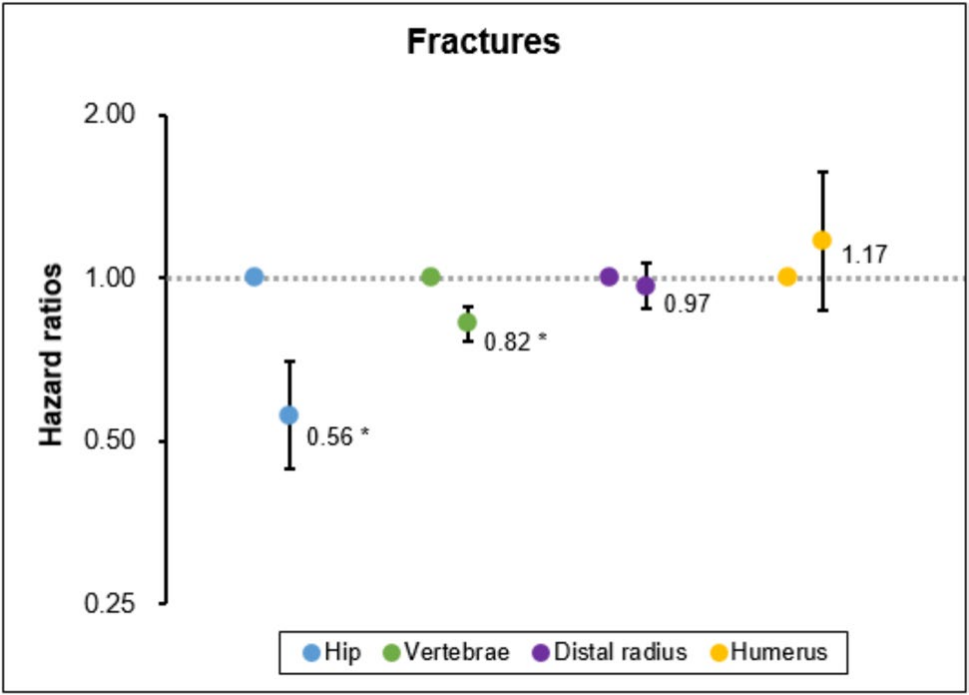
Results of analysis stratified by fracture site, Reference: screening without bone mineral density testing. Adjusted all covariates. *: *p*-value < 0.05

Figure 1 presents the results of additional analyses stratified by fracture site. The association was more pronounced for hip (HR: 0.56, 95% CI: 0.45–0.70) and vertebral (HR: 0.83, 95% CI: 0.77–0.89) fractures, with the strongest association observed for hip fractures. In contrast, no significant associations were found for distal radius or humerus fractures.

Table 4 shows the results of analyses according to time since fracture occurrence. Consistent with the pattern observed in Fig. 1, no significant association was found within 0–2 years or 2–5 years after screening; however, a stronger association emerged during the 5–10 year follow-up period (HR: 0.83, 95% CI: 0.77–0.90).

**Table 4:** Results of analysis stratified by follow-up time

Follow-up time	BMD testing	Fractures			
		HR	95% CI		
0–2 years	No	1.00			
	Yes	1.08	(0.94	-	1.24)
2–5 years	No	1.00			
	Yes	0.95	(0.86	-	1.06)
5–10 years	No	1.00			
	Yes	0.83	(0.77	-	0.90)

## Discussion

Our findings suggest that the inclusion of BMD testing in the Korean national health screening program was associated with increased osteoporosis-related medical visits and a subsequent reduction in the risk of osteoporotic fractures over a 10-year follow-up period. This association was particularly pronounced among individuals with a low BMI and was most evident for hip and vertebral fractures. Moreover, the preventive effect on fractures emerged over the long term following screening, indicating the potential benefit of early identification and management through population-based screening initiatives.

Several studies conducted in the United States, Canada, and Europe have demonstrated that osteoporosis screening strategies are not only cost-effective but also contribute to the prevention of subsequent fractures [22–25]. Although previous randomized trials evaluating the effectiveness of BMD testing as a screening strategy for fracture prevention have shown a slight reduction in fracture risk among those who received the test, the findings did not reach statistical significance [4, 26, 27]. Nevertheless, the USPSTF interpreted the overall body of evidence as indicating a meaningful association between BMD testing and reduced fracture risk, ultimately recommending its implementation in the general population [11]. Differences between the present findings and those of previous trials may be attributed not only to the randomized design of prior studies but also to variations in the selection of screened individuals based on primary fracture risk assessment, differences in follow-up duration (with the present study having a longer observation period), the broader age range of participants (65–90 years vs. only 66 years), and geographical differences between European countries and South Korea.

Previous studies attempting to infer the effectiveness of BMD testing as a screening tool in South Korea have primarily relied on cross-sectional data collected after its inclusion in the national health screening program [15, 28, 29]. These studies estimated the prevalence of osteoporosis and indirectly suggested the potential impact of BMD testing by showing that the proportion of physician-diagnosed osteoporosis was higher among individuals aged 66–68 years—the target age group for screening—than in other age groups [15]. Additionally, a cohort study demonstrated that individuals with osteopenia or osteoporosis, as identified through BMD testing, had a higher risk of fractures during a 10-year follow-up period than those with normal BMD, implying the predictive value of the screening program [16]. These findings align with the increased osteoporosis diagnoses observed. However, cross-sectional studies, which examine population prevalence trends, lack control groups, while cohort studies using post-BMD data from national screening programs focus on BMD accuracy, limiting their ability to directly assess the screening intervention's impact. Furthermore, unlike randomized controlled trials, which apply strict inclusion criteria, the direct population-based results of BMD testing introduction in this study might reflect the real-world screening environment [consider country-specific context [30].

Osteoporosis is often asymptomatic and may remain undiagnosed in the absence of screening [31, 32]. The increased detection of osteoporosis following the implementation of BMD testing highlights the role of screening in identifying previously unrecognized cases, thereby supporting its potential utility at the population level. However, findings from the screening program also revealed a gap between the identification of abnormal results and subsequent follow-up care. This suggests that the full impact of BMD testing on fracture prevention may be underestimated and could be enhanced through improved post-screening management. Recognizing one's fracture risk following diagnosis may prompt physician visits, clinical consultations, pharmacologic interventions, and lifestyle modifications to support bone health—all of which could contribute to reducing future fracture incidence [33–35].

However, existing evidence suggests that even among women diagnosed with osteoporosis, awareness of their condition does not necessarily lead to changes in health behaviors [35, 36]. This may explain the absence of effect modification across subgroups defined by health behavior in our study. Therefore, the observed association in the main analysis may reflect the pharmacologic effects in a subset of individuals who received treatment following screening, and such effects may have emerged over a relatively long period after the initial screening [37, 38]. Additionally, low BMI is a known risk factor for osteoporosis and has been associated with site-specific differences in fracture risk. Individuals with low BMI may have been more likely to perceive their susceptibility to osteoporosis following BMD testing, which could have prompted physician visits and clinical interventions. This heightened awareness and subsequent medical engagement may have contributed to the prevention of fractures, particularly hip fractures, which occur more frequently in individuals with low BMI [39, 40].

This study has strengths in evaluating the effectiveness of the national screening program using a population-based cohort and a natural policy change involving the introduction of BMD testing. The findings can also inform future screening strategies. However, several limitations should be noted. First, the claims data used in this study did not capture prescriptions that are not covered by insurance and may be subject to coding errors or patient misclassification. Second, BMD testing can be conducted through private health check-ups outside of the national screening program, and information regarding such testing was not available [41]. Since our study population was limited to individuals who participated in the national screening program, health-seeking behaviors may be distributed randomly between groups. Third, lifestyle factors such as alcohol consumption, smoking status, and physical activity were obtained through self-reported questionnaires, which are subject to recall bias. In particular, underreporting of smoking among women is a known concern in South Korea [42]. Fourth, the possibility of residual confounding due to unmeasured variables cannot be ruled out. Lastly, participants in our study may represent a healthier and more health-conscious population due to voluntary participation in health screening programs, potentially limiting the generalizability of our findings. In addition, extrapolation of the results to settings with different healthcare systems, to populations outside the age of 66 years, or to men should be undertaken with caution, and further research considering these conditions is warranted.

## Conclusion

The inclusion of BMD testing in South Korea’s national health screening program was demonstrated to enhance medical visits for osteoporosis and reduce the risk of subsequent osteoporotic fractures. This association was particularly pronounced among individuals with low BMI, highlighting the potential of BMD testing in preventing hip and vertebral fractures, which are more common in this population over the long term. Given that the current system may underestimate the full impact of BMD screening, strengthening post-screening management is essential to maximize its preventive benefits.

## Supplementary Information

Below is the link to the electronic supplementary material.

ESM 1 Supplementary Material 1 (DOCX 87.3 KB)

## Data Availability

The Korea National Health Insurance Service-National Sample Cohort (NHIS-NSC) is a public, open-access database. It is based on the health insurance claims data of all Koreans, and the sample cohort is available for public purposes and scientific research. The sample cohort data are available after approval for use by the National Health Insurance Service (https://nhiss.nhis.or.kr/bd/ab/bdaba000eng.do).

## Abbreviations

**BMD**: Bone mineral density
**NHIS-SC**: National Health Insurance Service–Senior Cohort
**CCI**: Charlson Comorbidity Index
**BMI**: Body mass index
**WHO**: World Health Organization
**OR**: Odds ratio
**HR**: Hazard ratio
**CI**: Confidence interval
