# Supplementary material for: Impact of bone mineral density testing in the national health screening program on osteoporosis-related medical visits and fractures among women
Source: Arch Osteoporos. 2026 Mar 25;21(1):56. doi: 10.1007/s11657-026-01673-1 (PMC13018048; doi:10.1007/s11657-026-01673-1)
Supplement: Supplementary file 1 — Supplementary Material 1 (DOCX 87.3 KB) [file 11657_2026_1673_MOESM1_ESM.docx]

| **Supplementary Table S1. Result of incidence rate ratio of fractures.** | | | | | | | | |  |
| --- | --- | --- | --- | --- | --- | --- | --- | --- | --- |
| **Variables** | **Number of patients** | **Fracture incidence** | **Person-year** | **Crude IR per 1,000 person-year** | **IRR** | **95% CI** | | |  |
|  |  |  |  |  |  |  |  |  |  |
| **BMD testing** |  |  |  |  |  |  |  |  |  |
| No | 10,566 | 2,419 | 94,191 | 25.7 | 1.00 |  |  |  |  |
| Yes | 14,329 | 2,923 | 128,812 | 22.7 | 0.88 | (0.84 | - | 0.93) |  |

IRR: incidence rate ratio. CI: confidence interval. BMD: bone mineral density.

| **Supplementary Table S2. Testing methods and distribution of bone mineral density results in the study population.** | | | |
| --- | --- | --- | --- |
| **BMD testing method** | **N (%)** | **BMD testing results (T-score)** | **N (%)** |
| Total | 12,598 (100) | Total | 12,934 (100) |
| Dual-energy X-ray absorptiometry (DXA) | 6,948 (55.2) | Normal (-1.0 or higher) | 2,134 (16.5) |
| Peripheral dual-energy X-ray absorptiometry (pDXA) | 1,248 (9.9) | Osteopenia (Between -1.0 and -2.5) | 5,172 (40.0) |
| Quantitative computed tomography (QCT / pQCT) * | 1,846 (14.7) | Osteoporosis (-2.5 or lower) | 5,628 (43.5) |
| Quantitative ultrasound (QUS) | 2,556 (20.3) |  |  |

*: Results of (peripheral) quantitative computed tomography (QCT or pQCT) method: normal (>120 mg/cm³), osteopenia (80–120 mg/cm³), and osteoporosis (<80 mg/cm³).


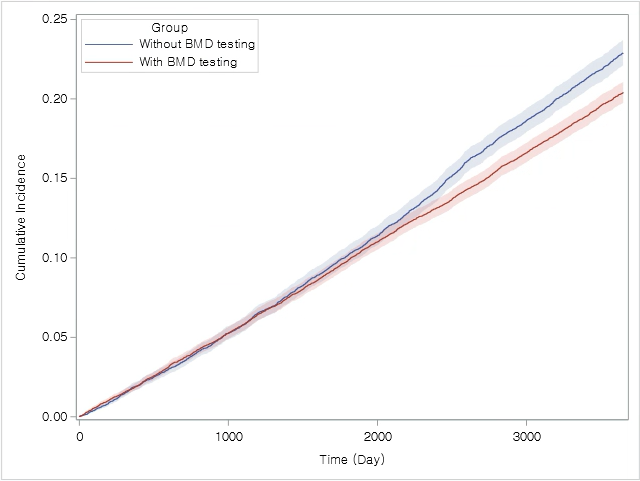


**Supplementary Figure S1. Cumulative incidence of fractures by inclusion of BMD testing in the national health screening program.**

Log-rank test <.0001
